# Interventions to prevent aspiration in older adults with dysphagia living in nursing homes: a scoping review

**DOI:** 10.1186/s12877-021-02366-9

**Published:** 2021-07-17

**Authors:** Shen Chen, Bridie Kent, Yan Cui

**Affiliations:** 1 School of Nursing, Nanjing Medical University, Nanjing, China; 2 The University of Plymouth Centre for Innovations in Health and Social Care: A Joanna Briggs Institute Centre of Excellence, Plymouth, UK; 3 School of Nursing and Midwifery, University of Plymouth, Plymouth, UK

**Keywords:** Aspiration, Aged, Resident, Dysphagia, Nursing homes, Scoping review

## Abstract

**Background:**

Dysphagia is highly prevalent condition in older adults living in nursing homes. There is also evidence indicating that aspiration is one of the major health risks for these older adults, which is more likely to result in respiratory infections, aspiration pneumonia and sudden bolus death. Evidence syntheses have demonstrated the effectiveness of interventions for prevention of aspiration among hospitalized older people. The aim of this scoping review is to describe the current spread of interventions to prevent or reduce aspiration in older adults with dysphagia with a specific focus on those who reside in nursing homes.

**Methods:**

The Joanna Briggs Institute methods and PRISMA-ScR guidelines were used to inform this review. MEDLINE, CINAHL, EMBASE, Cochrane Library, Joanna Briggs Institute EBP Database and Web of Science were searched for related articles from 2010 to 2020 as well as Chinese databases (CNKI, WANFANG DATA and VIP) and databases for unpublished material. A three-step search strategy was utilized, including the use of citation software to manage search results and de-duplication, abstract review and full-text review by two reviewers. Details of included studies were then extracted using a prepared data extraction tool. The resulting map was displayed in tabular form along with a narrative summary.

**Results:**

Although 637 articles were located, 19 papers were included in the final analysis. Interventions to prevent aspiration in older adults with dysphagia living in nursing homes included: more bedside evaluation, modification of dietary, creating an appropriate environment for swallowing, providing appropriate feeding assistance, appropriate posture or maneuver for swallowing, appropriate rehabilitation program, medication treatment, and stimulation treatment.

**Conclusion:**

Nursing homes, particularly those in developing countries, require more support for staff training and necessary equipment. Professional interventions provided by speech and language therapists are still limited in the setting of nursing homes. Modification of dietary was the most frequently used intervention to prevent or reduce aspiration. Multi-disciplinary interventions had the best results for aspiration management, but for many nursing homes, access to such teams is limited. Nursing home residents respond well to person-centered interventions that have a comprehensive consideration of their degree of aspiration risk, health condition, individual feelings and cognitive state.

## Background

Dysphagia, which is highly prevalent in old age, was recognized as a geriatric syndrome by the European Union Geriatric Medicine Society, who defined it as a condition involving perceived, or real, difficulty in forming, or moving, a bolus safely from the oral cavity to the esophagus [1, 2]. In short, it is a difficulty in swallowing. The prevalence of dysphagia in older adults, across different settings, has been calculated, with rates between 51 and 60% among institutionalized older adults, 44% in those admitted to geriatric acute care settings, and an average rate of 15% in the community dwelling older adults [1, 3–5]. According to these data, it is important to explore more fully the interventions that might help older adults who reside in institutions, such as nursing homes and residential care facilities, as they are more likely to suffer from dysphagia.

Most frequently, dysphagia is a result of altered physiology of deglutition caused by ageing, frailty, cancer of the neck and esophagus, or neurological diseases such as stroke, dementia and Parkinson disease [6–9]. Dysphagia is a serious condition that may lead to a decline in quality of life and is associated with many poor outcomes [10]. These include impaired swallowing efficacy, or the ineffective ingestion of nutrients and liquids, may cause malnutrition and/or dehydration [11, 12]. An impaired safety mechanism of swallowing, which can result in airway invasion, may lead to a complication called aspiration [11, 13, 14]. All these negative outcomes result in increased rates of hospitalization, hospital readmission, psychological distress and mortality [1, 14, 15].

Aspiration has been defined as the misdirection of oropharyngeal or gastric contents into the larynx and lower respiratory tract [16]. It is one of the major health risks for older adults with dysphagia. Through the use of the gold standard test for aspiration, Video Fluoroscopic Swallowing Study (VFSS), the prevalence of aspiration has been calculated to be between 43 and 51% in people with dysphagia [17, 18]. In addition, 55 to 59% of this population were diagnosed as having silent aspiration, where solids or liquids were aspirated into their airways with no cough or shortness of breath, because of impairment of the cough reflex [17, 18]. Such people are more likely to suffer from severe outcomes such as respiratory infections, aspiration pneumonia and sudden bolus death [11, 13, 14]. Previous studies showed that people who suffered long periods of time of aspiration had a significantly higher risk of death within 1 year than those with no aspiration [19–21]. Although the complications of dysphagia and aspiration can be severe, they are often undetected and untreated [22]. Therefore, it is very important and necessary to explore some interventions to avoid or reduce aspiration.

A preliminary review of the literature found that the scope of existing interventions to prevent aspiration in dysphagia people has not been fully captured. In particular, as nursing homes report a higher proportion of older adults with dysphagia than other settings, and they seldom have professional intervention equipment or similar ratios of healthcare staff to clients, when compared with hospitals, it was clear that, it was important to understand the extent of possible interventions suitable for implementation in nursing homes and residential care facilities. Therefore, this scoping review focused on all nursing homes settings and aimed to describe the current evidence on interventions to prevent or reduce aspiration in older adults with dysphagia who reside in these settings. By doing so, it may then help healthcare professionals and caregivers to identify the most appropriate approaches to avoid complications caused by aspiration and improve quality of life for older adults with dysphagia.

A peer reviewed protocol guided the implementation of this scoping review.

## Methods

The JBI methodology for scoping reviews [23] was used. Reporting guidelines Preferred Reporting Items for Systematic Reviews and Meta-Analyses extension for Scoping Reviews (PRISMA-SR) were adhered [24].

### Review question

What interventions are available to prevent or reduce aspiration in older adults with dysphagia who live in nursing homes or residential facilities?

### Inclusion criteria

#### Participants

This scoping review only included older adults with dysphagia who lived in nursing homes or residential facilities. Older adults were defined as people aged 60 years or older, as this standard is used by many developing countries, and can cover the standard of 65 years or older.

#### Concept

These included all interventions designed to prevent aspiration for older adults with dysphagia who reside in a nursing home setting. All types of interventions, intervention providers, target residents of intervention, locations of intervention, and results of intervention were included as concepts.

#### Context

The context included interventions that occurred in all countries and were provided by both healthcare professionals and/or formal or informal caregivers and limited to nursing homes or residential facilities.

#### Types of sources

In order to be inclusive, both experimental and observational quantitative studies including randomized controlled trials, non-randomized controlled trials, quasi-experimental, before and after studies, prospective and retrospective cohort studies, case control studies, and analytical and descriptive cross-sectional studies were considered for inclusion. This scoping review also considered qualitative designs such as phenomenology, grounded theory, ethnography, action research and feminist research.

#### Exclusion criteria

Exclusions were interventions for adults under 60 years of age and interventions provided for people with esophageal dysphagia or without dysphagia, such as an intervention designed for people with aspiration caused by gastroesophageal reflux. People being cared for in hospital, community day centers or their own homes were also excluded; as were non-English studies, with the exception of Chinese language. JBI Manual allows restrictions on source inclusion by language when there are feasibility reasons. Our authors only have the ability to read English and Chinese.

### Search strategy

A search strategy was developed, with assistance from an information scientist, and it aimed to find both published and unpublished studies over the last 10 years from 2010 to 2020, in order to capture the most up-to-date evidence. An initial limited search of MEDLINE and CINAHL using preliminary keywords was firstly undertaken to identify articles on the topic. The preliminary keywords include: dysphagia, swallowing disorder, swallowing difficulty, aspiration, airway invasion, nursing homes, residential care facilities, older adults, the elderly, intervention and management.

The databases to be searched for published material include: MEDLINE, CINAHL, EMBASE, Cochrane Library, Joanna Briggs Institute EBP Database and Web of Science. The search terms included MeSH and “free text” terms in combination. Chinese databases included: CNKI (https://www.cnki.net/), WANGFAN DATA (http://www.wanfangdata.com.cn/index.html) and VIP (http://qikan.cqvip.com/). The databases for unpublished material included Open Grey, British Library, CADTH, EThOS, MedNar, TRIP Database and Google Scholar.

The text words contained in the titles and abstracts of relevant articles, and the index terms used to describe the articles were extracted. A full secondary search was then performed using the terms identified from the initial review results. Full search strategy is provided in **Appendix 1**. References from retrieved articles were then searched for additional studies for the final stage of the process.

### Title and abstract screening

Citation management software EndNote X9 (Clarivate Analytics, PA, USA) was used to manage the list of all citations retrieved and all unnecessary duplicate records were eliminated. Two independent reviewers screened the title and abstract of each article to determine its relevance to the inclusion criteria. Potentially relevant studies were retrieved in full and their citation details imported into the Joanna Briggs Institute System for the Unified Management, Assessment and Review of Information (JBI-SUMARI; JBI, Adelaide, Australia). The full text of selected citations was assessed in detail against the inclusion criteria by two independent reviewers. Reasons for exclusion were documented. Any disagreements that arise between the reviewers at each stage of the study selection process were resolved through discussion.

### Data extraction

A modified JBI data extraction tool was developed by the reviewers; the extraction tool is listed in **Appendix 2**. The data extracted included specific details about the population, concept, context, study methods and key findings relevant to the review objective such as: types of interventions, intervention providers, target residents of intervention, locations of intervention, results of intervention.

## Results

**Fig. 1:**
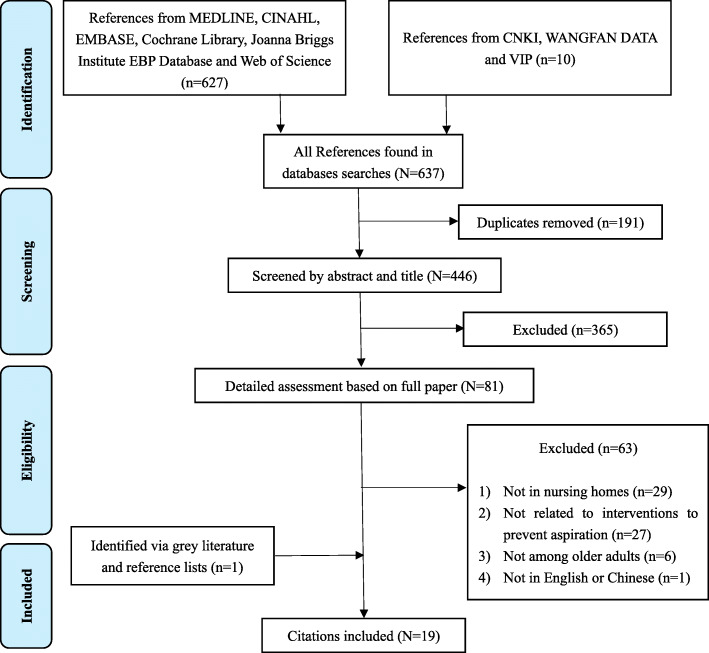
Flow chart of study selection

The literature search resulted in a total of 446 citations after duplicates were removed. The titles and abstracts for these citations were screened and 81 citations were considered for further detailed assessment of the full paper. After further screening, 29 were excluded for not being in settings of nursing homes, 27 were excluded for not being related to interventions to prevent aspiration, 6 were excluded for not being studies conducted among older adults, and 1 was excluded for not being written in English or Chinese. Subsequently, 19 papers were identified for data extraction, including one of which was identified via grey literature and reference lists. A flow chart showing the number of citations at each stage is described in Fig. 1.

### Country or region for publication

As Table 1 shows, of the nineteen studies [13, 25–42], six were conducted in the USA, three were conducted in Japan, two were conducted in Australia, and one was conducted in the UK, France, Sweden, Brazil, South Korea, Mainland China, Hongkong and Taiwan, respectively. Most were conducted in developed countries or regions (89.5%).

**Table 1:** Summary of studies

Authors (Year)	Study design	Setting	Country or region	Target residents of intervention	Intervention providers
Cormary X, et al. (2018) [25]	Quasi-experimental Study	Nursing home	France	Frail residents	Nurses, speech therapists, doctors, dentists, dieticians, a 3-star chef and catering managers
Freiry AM, et al. (2017) [26]	Case series	Long stay institution	Brazil	Dementia residents	Speech-language therapists
Gilmore-Bykovskyi AL, Rogus-Pulia N. (2018) [27]	Observational study	Nursing Home	USA	Dementia residents	Nursing assistants
Gokula M, et al. (2011) [28]	Case series	Long-term care facility	USA	Residents with prior stroke, Parkinson’s disease or swallowing problems related to aging	Long-term care facility staff
Hagglund P, et al. (2019) [29]	Randomized controlled trial	Intermediate care unit	Sweden	Residents with swallowing dysfunction	Calibrated professionals (registered dental hygienists and speech-language pathologist)
Hajjar S, Wollman D. (2019) [30]	Case report	Nursing home	USA	Residents with Parkinson’s disease presented with aspiration pneumonia	Caregivers
Kuramoto N, et al. (2018) [31]	Quasi-experimental Study	Nursing home	Japan	Nursing home residents	Caregivers
Levenson SA, Walker VL. (2019) [32]	Literature review	Nursing home	USA	Nursing home residents	Caregivers
Luk JKH, Chan DKY. (2014) [33]	Literature review	Residential care home	Hong Kong	Nursing home residents	Caregivers
Mesioye A, et al. (2018) [34]	Quasi-experimental Study	Veteran’s community living center	USA	Residents who were receiving modified diets or required feeding assistance	Interdisciplinary team
Morley JE. (2015) [13]	Literature review	Nursing home	USA	Nursing home residents	Nurses and caregivers
Hines S, et al. (2010) [35]	Systematic review	Residential aged care facility	Australia	Dementia residents	Caregivers
Painter V, et al. (2017) [36]	Systematic review	Residential aged care facility	Australia	Dementia residents	Caregivers
Park Y, et al. (2015) [37]	Pseudo Randomized Controlled Trial	Nursing home	South Korea	Nursing home residents	Geriatric care workers, social workers and nurses
Richards E. (2012) [38]	Literature review	Nursing home	UK	Residents with swallowing and communication difficulties after stroke	Speech-language therapists and nursing home staff
Yamada T, et al. (2017) [39]	Pseudo Randomized Controlled Trial	Nursing home	Japan	Nursing home residents	Researchers
Lu M, et al. (2018) [40]	Pseudo Randomized Controlled Trial	Nursing home	China	Residents with dysphagia	Nurses and general practitioners
Chiang CK, Hwu YJ. (2018) [41]	Qualitative study	Long-term care facility	Taiwan	Nursing aides	Nursing aides
Takamoto K, et al. (2017) [42]	Randomized Controlled Trial	Elder care facility	Japan	Nursing home residents	The facility staff and experimenters

### Study design

As Table 1 shows, only one study used a qualitative design [41], and the topic was related to feeding experiences from nursing aides. Two studies were randomized controlled trials [29, 42]. Three were classified as pseudo randomized controlled trials and another three were quasi-experimental studies [25, 31, 34, 37, 39, 40]. Observational design was used in one study [27]. Of the six reviews, four were literature reviews and the other were systematic reviews [13, 32, 33, 35, 36, 38]. A further two studies utilized case series approaches and one was case report [26, 28, 30].

### Target residents of intervention

As Table 1 shows, one study reported its targets of intervention are frail residents [25]. Several studies included residents with specific diseases as intervention targets. Two studies mentioned Parkinson’s disease [28, 30], another two included post-stroke older adults [28, 38] and a further four included residents with dementia [26, 27, 35, 36]. Only one study included residents with swallowing problems related to aging [28]. Eleven studies introduced their targets of intervention as residents with varying degrees of swallowing problems, but did not report the causes of dysphagia [13, 29, 31–34, 37, 39–42].

### Intervention providers

As Table 1 shows, five studies used an interdisciplinary team as their intervention providers [25, 34, 37, 38, 40], which meant that residents with dysphagia experienced multiple interventions from two or more kinds of professionals. Five studies reported their intervention providers to be nurses [13, 25, 34, 37, 40], two included nursing assistants or aides [27, 41], and five included speech-language therapists [25, 26, 29, 34, 38]. In addition, nine studies only indicated that the interventions were conducted by nursing home staff without clarifying their specific occupations [28, 30–33, 35, 36, 39, 42].

### Interventions

**Fig. 2:**
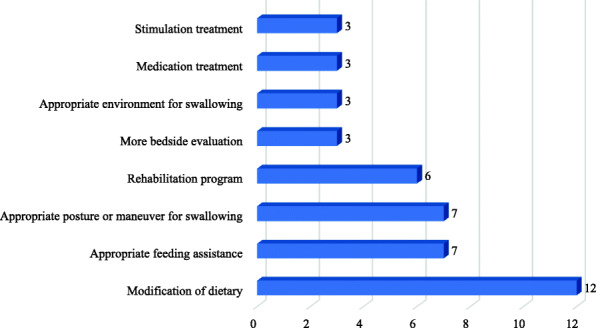
Frequency of reporting of interventions

As Table 2 shows, the interventions from included studies can be classified into eight groups. As Fig. 2 indicates, modification of dietary, appropriate feeding assistance, appropriate posture or maneuver for swallowing and rehabilitation program were the most frequently reported among all included studies.

**Table 2:** Interventions and the articles that refer to them

Authors (Year)	More bedside evaluation	Modification of dietary	Appropriate environment for swallowing	Appropriate feeding assistance	Appropriate posture or maneuver for swallowing	Rehabilitation program	Medication treatment	Stimulation treatment
Cormary X, et al. (2018) [25]	*			*	*			
Freiry AM, et al. (2017) [26]						*		
Gilmore-Bykovskyi AL, Rogus-Pulia N. (2018) [27]				*				
Gokula M, et al. (2011) [28]							*	
Hagglund P, et al. (2019) [29]						*		
Hajjar S, Wollman D. (2019) [30]		*						
Kuramoto N, et al. (2018) [31]					*			
Levenson SA, Walker VL. (2019) [32]		*						
Luk JKH, Chan DKY. (2014) [33]		*	*		*		*	*
Mesioye A, et al. (2018) [34]	*	*	*				*	
Morley JE. (2015) [13]		*		*	*			*
Hines S, et al. (2010) [35]		*						
Painter V, et al. (2017) [36]		*						
Park Y, et al. (2015) [37]	*	*		*	*	*		
Richards E. (2012) [38]		*		*	*	*		*
Yamada T, et al. (2017) [39]		*						
Lu M, et al. (2018) [40]		*	*	*		*		
Chiang CK, Hwu YJ. (2018) [41]		*		*	*			
Takamoto K, et al. (2017) [42]						*

#### More bedside evaluation

Conducting more bedside evaluation was mentioned in three studies to avoid some inappropriate care delivered by caregivers during mealtime. Thus, nursing home staff screened the risk of complication, evaluated the individualized menu and laminated cards kept in the dining areas, grouped by the degree of dysphagia risk, and then gave a quick check on symptoms and signs such as coughing, drooling, making a grunting sound during mealtime [25, 34, 37].

#### Modification of dietary

Twelve studies reported that modification of dietary consistency or texture was a commonly used method in nursing homes to prevent or reduce aspiration [13, 30, 32–41]. Such modification usually involved the use of thickening agents to change the consistency of food or fluids. Of these studies, seven used this intervention as a part of an interdisciplinary prevention plan [13, 33, 34, 37, 38, 40, 41]; the others focused on it to explore its independent effects [30, 32, 35, 36, 39]. Most of studies concluded that texture-modified food and liquids reduced the risk of aspiration, and that this was helpful in maintaining adequate fluid intake for residents with dysphagia. However, Levenson reported that no clear correlation was found between viscosity and successful prevention of aspiration pneumonia [32]. Painter also reported that food consistency change was useful in reducing the risk of aspiration seen on video fluoroscopy, but little evidence supported the use of texture-modified food to improve the clinical consequence of aspiration pneumonia [36]. In addition, Richards reported that strengthening taste, such as by using sour drink or food, was helpful in improving the delayed onset of oral movement and swallow, which might be a useful strategy to reduce the risk of aspiration [38].

#### Appropriate environment for swallowing

Three studies reported that the eating environment might influence the risk of aspiration. Luk suggested that nursing homes should provide a quiet environment without distraction during mealtime [33]. Mesioye reported that environmental factors, such as dining room lighting, should be considered [34]. Lu advised that appropriate tableware is also very important [40].

#### Appropriate feeding assistance

Seven studies reported that using appropriate skills and equipment to assist residents to have meals could reduce the risk of aspiration. The skills included behaviors such as feeding slowly and adjusting the rate to the resident’s pace, modifying the volume of bolus or fluid when feeding, direct eye-contact, showing approval, orientation, providing choices, asking the resident for cooperation and giving appropriate emergency treatment for aspiration [13, 25, 27, 37, 38, 40, 41]. Regarding the equipment, it was only mentioned that appropriate adaptive equipment should be available, but no specific equipment was reported [13, 25].

#### Appropriate posture or maneuver for swallowing

Seven studies suggested that using appropriate postures or maneuvers could prevent aspiration when eating or drinking [13, 25, 31, 33, 37, 38, 41]. For those who can get out of bed, caregivers should help them sit upright when eating [33]. For those who are not able to get out of bed, it is important to raise the head of the bed by at least 30 degrees [33]. Tucking the chin toward the chest, which has also been called chin-tuck or chin-down, can prevent bolus or liquid from entering the airway [13, 33, 38]. Turning the head towards the paralyzed side and tilting the body to the non-paralyzed side is recommended as these actions makes it easier and safer to direct the food or fluid bolus downward and pass through the weaker side [33, 38]. In order to inform residents about an appropriate posture adjustment, Kuramoto developed a smartphone-based neck worn swallowing monitor, which was found to enhance safer mealtime assistance with less risk of aspiration [31].

Some swallowing maneuvers are also useful in strengthening swallowing function and preventing or reducing aspiration. Luk and Richards suggested that caregivers should guide residents to use some or all of the following: double or multiple swallowing, hard swallowing, effortful swallowing, repeated head lift maneuver, supraglottic swallowing (technique to hold breath, then swallow, then cough), Masako maneuver (holding tongue in teeth and dry swallowing) and Mendelsohn maneuver (prolonging upper position of larynx during swallow) [33, 38].

#### Swallowing rehabilitation program

Six studies used different kinds of swallowing rehabilitation programs to reduce aspiration by improving swallowing function [26, 29, 37, 38, 40, 42]. Freiry conducted a four-week cryotherapy therapy and found that all participants improved their gag reflex sensitivity and reduced reflex swallowing time, indicating that the risk of aspiration was decreased [26]. Hagglund provided neuromuscular training of the pharyngeal and orofacial muscles for 5 weeks. At the end of the training period, aspiration signs were significantly reduced in the intervention group when compared with control group [29]. Takamoto guided participants to have a four-week lip closure training, and found the training was useful in reducing the risk of pulmonary aspiration [42]. The other three studies reported that tongue and oral exercises, neck muscle exercises, pursed-lip breathing, coughing exercises, oromotor exercises and vocal adduction exercises were effective in improving functional deglutition and enhancing impaired swallowing physiology. Furthermore, the risk of aspiration improved significantly after these rehabilitation programs [37, 38, 40].

#### Medication treatment

Three studies reported that specific medications benefitted the swallowing ability of residents with dysphagia [28, 33, 34]. Gokula administered a 50 mg to 100 mg daily dose of amantadine to 12 residents with swallowing problems. After using for 2 to 4 weeks, 11 patients demonstrated decreased cough while eating. After 4 to 6 weeks of use, these residents could gradually tolerate food without aspiration [28]. Luk introduced more medications which were able to improve the swallowing reflex, such as amantadine, levodopa, cilostazol, folate and angiotensin-converting enzyme inhibitors [33]. The author emphasized that it may be beneficial for older adults with dysphagia to continuously use angiotensin-converting enzyme inhibitor, if they have no intolerable cough [33]. When introducing the Interdisciplinary collaboration, Mesioye commented that physicians or pharmacists need to carefully review whether residents are taking medications that aggravate swallowing disorders [34].

#### Stimulation treatment

Of all the included studies, only three, which were literature reviews, reported that some neurostimulation approaches could reduce the risk of aspiration by improving swallowing function, based on the theory that thermal or mechanical stimulation at the anterior oropharynx can bring about reflex swallowing action [13, 33, 38]. These approaches included neuromuscular electrical stimulation, thermal tactile stimulation, repetitive transcranial magnetic stimulation to directly stimulate the pharyngeal motor cortex, and other chemical, physical or electrical stimulation to stimulate the peripheral oropharyngeal sensory system [13, 33, 38]. Previously, these approaches used to be only performed in hospitals, but with the upgrading and miniaturization of equipment, it is now possible that stimulation treatments can also be carried out in nursing homes [13].

## Discussion

This scoping review included nineteen studies, with the majority conducted in developed countries or regions. There is a dearth of evidence from low to middle income countries, possibly because long-term care facilities in such countries are still at their early stage of development [43]. For example, China, the country with the largest ageing population in the world, only had approximately 7.5 million beds in nursing homes of the entire country [44]. Although the studies included in this review reported that their interventions were useful in preventing or reducing aspiration, theses intervention may not always suitable to be applied in developing countries, as these countries may not have sufficient resources such as well-trained nursing home staff and the necessary equipment [44].

Speech and language therapists (SLTs) perform an important role in the predominant management of dysphagia [26]. However, only five studies reported that their intervention providers included SLT teams [25, 26, 29, 34, 38]. Without the input of SLTs, nursing home may not be able to resolve some complicated problems related to their residents’ swallowing function, and nursing home caregivers may not have the appropriate knowledge and skills to provide professional interventions for residents with dysphagia to reduce the risk of aspiration [44]. Limited by economic conditions, many nursing homes in developing countries are unable to hire SLT teams [44]. In many nursing homes, nurses are the sole registered health professionals, whilst in other residential care facilities, nursing assistants and other unregistered caregivers provide the essential care [45]. Therefore, residents with dysphagia in these institutions may experience repeated aspiration, and then suffer from severe aspiration pneumonia.

Modification of diet as an intervention was reported most frequently among all included studies, and it has been widely applied in many residential care facilities [46]. However, it is also one of the most controversial interventions. Few studies applied randomized controlled trail design and used video fluoroscopy or fiberoptic endoscopy to determine that the modification of diet can successfully prevent or reduce aspiration. Therefore, it has been questioned whether it is a reliable and justified intervention and whether it is necessarily better, as there is no strong evidence to show such methods reduce pneumonia [32, 36, 47]. It has been reported that modified diets and thickened liquids were often less palatable, and their texture and taste were strongly disliked by the residents [13, 32, 33]. Malnutrition, dehydration, sub-therapeutic medication levels and reduced quality of life were common adverse consequences caused by modification of diet [13, 32, 33, 36]. Luk also indicated that liquids thickener is expensive, and blending and pureeing are labor-intensive [33]. Moreover, Carlisle reported that starch-thickened liquids had dangerous interaction with polyethylene glycol 3350 laxative (PEG) [48]. A precipitous loss of thickening happened when PEG was put into starch-based thickeners [48]. Therefore, if residents are using PEG, starch-based thickeners should be avoided when modifying diet. In addition, Ebihara reported that the reflex of swallowing was delayed seriously at body temperature [49]. Therefore, meals should be prepared immediately prior to eating, and pre-made food should be properly heated.

Using specific postures or maneuvers was also a popular intervention in many studies. They are very easy to learn and do not need great efforts [50]. However, many older adults who have cognitive impairment cannot follow such instructions of chin-tuck or chin-down [51, 52]. Swallowing postures and maneuvers do not always work for older people who suffer with dysphagia. Chin-tuck or chin-down was shown to help avoid aspiration in about 55% of cases, whilst a head-turned posture only worked for 25.3% [50, 51]. The effectiveness of chin-tuck, chin-down and head-turned posture are not good enough to prevent aspiration. Hence, aspiration may still happen frequently when using these approaches.

To prevent or reduce aspiration, a single intervention is often not enough. Many studies used an interdisciplinary team to provide, for residents with dysphagia, a multi-disciplinary prevention plan [25, 34, 37, 38, 40]. Direct interventions such as diet modification and chin-tuck or chin-down posture, which are usually used during the feeding process, are very easy and convenient to apply [1]. However, they are not the permanent solution. Indirect interventions such as rehabilitation programs that focus on exercising specific muscles or muscle groups, stimulation treatment and pharmacological treatment can reduce aspiration by improving swallowing function [53–56], although there is a case series questioned the effect of thermal-tactile stimulation [57]. But they usually take a long time with slow effects, and they cannot be used alone in the absence of direct interventions. Oral care has also been indicated as an indirect intervention that can help prevent aspiration pneumonia [58]. However, oral care is an adjuvant intervention and one that all older people should receive. Interdisciplinary interventions have a huge advantage in aspiration management. Based on the included studies, the interdisciplinary team usually contained part of or all of the following members: speech and language therapists, doctors, dentists, nurses, nursing assistants, care workers, social workers, dieticians, chefs and catering managers [25, 34, 37, 38, 40]. Consequently, the costs are high for interventions provided by so many specialists and it may be unacceptable for residents, especially those in developing countries, to pay a lot of money to acquire the multi-disciplinary services [44]. Thus, when providing intervention services, the feelings, needs and payment levels of residents should also be taken into account by nursing homes.

In this review, only two studies were standard randomized controlled trials and another two were systematic review. Therefore, many studies did not use high quality study designs, which may cause some bias and limit the reliability of the conclusion that their interventions were useful.

Many of existing interventions do not appear to be suitable for residents who have dysphagia and cognitive impairment. Such people find is much harder to follow the instructions from intervention providers [50, 51]. Feeding tubes are frequently used to ensure enough nutrition intake, but these are not the preferred option. For preventing aspiration in residents with dementia or other forms of cognitive impairment [13]. Therefore, more suitable interventions targeting residents with cognitive impairment are urgently needed.

The main limitation of this review is not including studies that were published in languages other than English and Chinese, which may cause publish bias as quantities of articles were published in other language. For future, this scoping review can perform as a precursor to several systematic reviews that focus on some specific interventions.

## Conclusion

The majority of the included studies were conducted in developed countries or regions. The review provides a useful overview of interventions that have been used in nursing or residential facilities with older people who suffer from dysphagia, but the dearth of evidence from low to middle income countries is noticeable. Professional interventions provided by speech and language therapists are still limited in the setting of nursing homes. Modification of diet was the most frequently used intervention to prevent or reduce aspiration, however, multi-disciplinary interventions were far better for aspiration management. Ideally residents should be offered person-centered interventions that have a comprehensive consideration of their degree of aspiration risk, health condition, individual feelings and cognitive state.

## Data Availability

Our data or material may be available from corresponding author or first author upon reasonable request.

## Appendix 1

### Search strategy

Search date: 02 May 2020.

## 1. Search strategy for MEDLINE (Ovid)

**Table:**

Search number	Search terms	Results
1	exp Deglutition Disorders/	52,073
2	(dysphagi* or orophargngeal dysphagia or esophageal dysphagia or deglutition disorder* or swallowing disorder* or swallowing dysfunction or swallowing problem* or swallowing difficult* or difficulty in swallowing or swallowing disabilit*).mp	37,864
3	1 or 2	67,773
4	exp Pneumonia, Aspiration/	5967
5	exp Respiratory Aspiration/	1412
6	exp Inhalation/	5645
7	(aspiration or penetration or airway invasion or aspiration pneumonia* or silent aspiration or aspirator or choking).mp	153,150
8	4 or 5 or 6 or 7	157,920
9	exp Aged/	3,082,483
10	exp “Aged, 80 and over”/	899,385
11	(older persons or older adults or older people or older patients or geriatric* or aged or aging or older or elder or elderly or the elderly or elderly people or senior*).mp	5,679,572
12	9 or 10 or 11	5,679,572
13	exp Early Medical Intervention/	2920
14	exp Disease Management/	68,664
15	exp Rehabilitation/	300,959
16	exp Rehabilitation Nursing/	1400
17	exp Therapeutics/	4,535,604
18	(intervention or management or treatment or rehabilitation or therapy or strateg* or program* or practice* or prevent* or control or method* or texture modif* or bolus modif* or diet modif* or food modif* or fluids modif* or viscosity modif* or consistency modif* or texture adapt* or thicken* or swallow* maneuver* or swallow* posture* or chin down or chin tuck or double deglutition or hard swallow* or stimulation).mp	16,704,201
19	13 or 14 or 15 or 16 or 17 or 18	17,494,545
20	exp Residential Facilities/	51,952
21	exp Nursing Homes/	38,848
22	(nursing home* or residential care facilit* or residential facilit* or aged care facilit* or elderly care facilit* or long-term care facilit* or care home or institution*).mp	345,004
23	20 or 21 or 22	354,267
24	3 and 8 and 12 and 19 and 23	188
25	Limit 24 to yr = “2010-Current”	96

## 2. Search strategy for EMBASE (Ovid)

**Table:**

Search number	Search terms	Results
1	exp dysphagia/	71,426
2	(dysphagi* or orophargngeal dysphagia or esophageal dysphagia or deglutition disorder* or swallowing disorder* or swallowing dysfunction or swallowing problem* or swallowing difficult* or difficulty in swallowing or swallowing disabilit*).mp	80,527
3	1 or 2	83,487
4	exp food aspiration/ or exp. aspiration/ or exp. aspiration pneumonia/ or exp. pulmonary aspiration/ or exp. liquid aspiration/	52,547
5	exp inhalation/	24,427
6	(aspiration or penetration or airway invasion or aspiration pneumonia* or silent aspiration or aspirator or choking).mp	283,322
7	4 or 5 or 6	306,853
8	exp aged/	2,945,786
9	(older persons or older adults or older people or older patients or geriatric* or aged or aging or older or elder or elderly or the elderly or elderly people or senior*).mp	5,026,418
10	8 or 9	5,026,418
11	exp nursing intervention/ or exp. early intervention/	26,116
12	exp nursing management/ or exp. disease management/ or exp. health care management/ or exp. medication therapy management/	3,712,020
13	exp rehabilitation nursing/ or exp. rehabilitation/	384,593
14	exp therapy/	8,367,069
15	(intervention or management or treatment or rehabilitation or therapy or strateg* or program* or practice* or prevent* or control or method* or texture modif* or bolus modif* or diet modif* or food modif* fluids modif* or viscosity modif* or consistency modif* or texture adapt* or thicken* or swallow* maneuver* or swallow* posture* or chin down or chin tuck or double deglutition or hard swallow* or stimulation).mp	21,648,694
16	11 or 12 or 13 or 14 or 15	22,682,715
17	exp residential home/ or exp. home for the aged/	17,582
18	exp nursing home/	51,106
19	(nursing home* or residential care facilit* or residential facilit* or aged care facilit* or elderly care facilit* or long-term care facilit* or care home or institution*).mp	527,672
20	17 or 18 or 19	535,977
21	3 and 7 and 10 and 16 and 20	347
22	limit 21 to yr = “2010-Current”	249

## 3. Search strategy for CINAHL (EBSCO)

**Table:**

Search ID	Search terms	Results
S1	(MH “Deglutition Disorders”)	8910
S2	dysphagi* or orophargngeal dysphagia or esophageal dysphagia or deglutition disorder* or swallowing disorder* or swallowing dysfunction or swallowing problem* or swallowing difficult* or difficulty in swallowing or swallowing disabilit*	14,200
S3	S1 OR S2	14,200
S4	(MH “Pneumonia, Aspiration”)	1704
S5	(MH “Aspiration”)	2486
S6	aspiration or penetration or airway invasion or aspiration pneumonia* or silent aspiration or aspirator or choking	32,218
S7	S4 OR S5 OR S6	32,218
S8	(MH “Aged+”)	872,995
S9	(MH “Aged, 80 and Over+”)	307,528
S10	older persons or older adults or older people or older patients or geriatric* or aged or aging or older or elder or elderly or the elderly or elderly people or senior*	1,150,305
S11	S8 OR S9 OR S10	1,150,310
S12	(MH “Early Intervention+”) OR (MH “Nursing Interventions”)	29,346
S13	(MH “Nursing Management+”) OR (MH “Eating Disorders Management (Iowa NIC)”) OR (MH “Disease Management+”)	37,756
S14	(MH “Rehabilitation+”) OR (MH “Rehabilitation, Geriatric”) OR (MH “Rehabilitation Nursing”)	309,872
S15	(MH “Therapeutics+”)	1,603,337
S16	intervention or management or treatment or rehabilitation or therapy or strateg* or program* or practice* or prevent* or control or method* or texture modif* or bolus modif* or diet modif* or food modif* or fluids modif* or viscosity modif* or consistency modif* or texture adapt* or thicken* or swallow* maneuver* or swallow* posture* or chin down or chin tuck or double deglutition or hard swallow* or stimulation	4,546,938
S17	S12 OR S13 OR S14 OR S15 OR S16	4,879,368
S18	(MH “Nursing Homes+”)	29,768
S19	(MH “Residential Facilities+”)	34,498
S20	nursing home* or residential care facilit* or residential facilit* or aged care facilit* or elderly care facilit* or long-term care facilit* or care home or institution*	213,983
S21	S18 OR S19 OR S20	216,938
S22	S3 AND S7 AND S11 AND S17 AND S21	113
S23	S22 Limiters - Published Date: 20100101–20,200,531	77

## 4. Search strategy for Cochrane library

**Table:**

Search number	Search terms	Results
#1	MeSH descriptor: [Deglutition Disorders] explode all trees	2803
#2	dysphagi* or “orophargngeal dysphagia” or “esophageal dysphagia” or deglutition NEXT disorder* or swallowing NEXT disorder* or “swallowing dysfunction” or swallowing NEXT problem* or swallowing NEXT difficult* or “difficulty in swallowing” or swallowing NEXT disabilit*	4699
#3	#1 or #2	6539
#4	MeSH descriptor: [Pneumonia, Aspiration] explode all trees	330
#5	MeSH descriptor: [Respiratory Aspiration] explode all trees	363
#6	MeSH descriptor: [Inhalation] in all MeSH products	319
#7	aspiration or penetration or “airway invasion” or aspiration NEXT pneumonia* or “silent aspiration” or aspirator or choking	11,840
#8	#4 or #5 or #6 or #7	12,131
#9	MeSH descriptor: [Aged] in all MeSH products	8819
#10	MeSH descriptor: [Aged, 80 and over] explode all trees	2354
#11	“older persons” or “older adults” or “older people” or “older patients” or geriatric* or aged or aging or older or elder or elderly or “the elderly” or “elderly people” or senior*	535,020
#12	#9 or #10 or #11	535,020
#13	MeSH descriptor: [Early Medical Intervention] explode all trees	367
#14	MeSH descriptor: [Disease Management] explode all trees	4340
#15	MeSH descriptor: [Rehabilitation] explode all trees	34,236
#16	MeSH descriptor: [Rehabilitation Nursing] explode all trees	54
#17	MeSH descriptor: [Therapeutics] explode all trees	298,062
#18	intervention OR management OR treatment OR rehabilitation OR therapy OR strateg* OR program* OR practice* OR prevent* OR control OR method* OR texture NEXT modif* OR bolus NEXT modif* OR diet NEXT modif* OR food NEXT modif* OR fluids NEXT modif* OR viscosity NEXT modif* OR consistency NEXT modif* OR texture NEXT adapt* OR thicken* OR swallow* NEXT maneuver* OR swallow* NEXT posture* OR “chin down” OR “chin tuck” OR “double deglutition” OR hard NEXT swallow* OR stimulation	1,358,321
#19	#13 or #14 or #15 or #16 or #17 or #18	1,367,759
#20	MeSH descriptor: [Residential Facilities] explode all trees	1706
#21	MeSH descriptor: [Nursing Homes] explode all trees	1309
#22	nursing NEXT home* OR residential NEXT care NEXT facilit* OR residential NEXT facilit* OR aged NEXT care NEXT facilit* OR elderly NEXT care NEXT facilit* OR long-term NEXT care NEXT facilit* OR “care home” OR institution*	28,203
#23	#20 OR #21 OR #22	28,369
#24	#3 AND #8 AND #12 AND #19 AND #23	41
#25	#24 with Cochrane Library publication date from Jan 2010 to May 2020	31

## 5. Search strategy for web of science

**Table:**

Search ID	Search terms	Results
#1	TOPIC: (dysphagi* or orophargngeal dysphagia or esophageal dysphagia or deglutition disorder* or swallowing disorder* or swallowing dysfunction or swallowing problem* or swallowing difficult* or difficulty in swallowing or swallowing disabilit*)	33,132
#2	TOPIC: (aspiration or penetration or airway invasion or aspiration pneumonia* or silent aspiration or aspirator or choking)	303,245
#3	TOPIC: (older persons or older adults or older people or older patients or geriatric* or aged or aging or older or elder or elderly or the elderly or elderly people or senior*)	4,239,844
#4	TOPIC: (intervention or management or treatment or rehabilitation or therapy or strateg* or program* or practice* or prevent* or control or method* or texture modif* or bolus modif* or diet modif* or food modif* or fluids modif* or viscosity modif* or consistency modif* or texture adapt* or thicken* or swallow* maneuver* or swallow* posture* or chin down or chin tuck or double deglutition or hard swallow* or stimulation)	23,213,334
#5	TOPIC: (nursing home* or residential care facilit* or residential facilit* or aged care facilit* or elderly care facilit* or long-term care facilit* or care home or institution*)	687,070
#6	#5 AND #4 AND #3 AND #2 AND #1	213
#7	#6 Refined by: PUBLICATION YEARS: (2020 OR 2013 OR 2019 OR 2012 OR 2018 OR 2011 OR 2017 OR 2010 OR 2016 OR 2015 OR 2014)	142

## Appendix 2

**Table 3:** Data extraction tool

Reviewer	
Date	
**Scoping review details**	
Scoping review title	
Review objective/s	
Review question/s	
**Inclusion/exclusion criteria**	
Population	
Concept	
Context	
**Study details and characteristics**	
Author(s)	
Year	
Title	
Journal	
Study design	
Country/Region	
Aim/Purpose	
Setting	
Participants	
Sample	
**Details/results extracted from study**	
Types of intervention	
Intervention providers	
Target residents of intervention	
Locations of intervention	
Results of intervention	
Other key findings	

## Abbreviations

VFSS: Video Fluoroscopic Swallowing Study
PRISMA-SR: Systematic Reviews and Meta-Analyses extension for Scoping Reviews
JBI: Joanna Briggs Institute
JBI-SUMARI: Joanna Briggs Institute System for the Unified Management, Assessment and Review of Information
SLTs: Speech and language therapists
PEG: Polyethylene glycol 3350 laxative

## References

[1] Baijens LW, Clavé P, Cras P, Ekberg O, Forster A, Kolb GF (2016). European society for swallowing disorders – European Union geriatric medicine society white paper: oropharyngeal dysphagia as a geriatric syndrome. Clin Interv Aging.

[2] Cook IJ, Kahrilas PJ (1999). AGA technical review on management of oropharyngeal dysphagia. Gastroenterology.

[3] Madhavan A, LaGorio LA, Crary MA, Dahl WJ, Carnaby GD (2016). Prevalence of and risk factors for dysphagia in the community dwelling elderly: a systematic review. J Nutr Health Aging.

[4] Park YH, Han HR, Oh BM, Lee J, Park JA, Yu SJ, Chang HK (2013). Prevalence and associated factors of dysphagia in nursing home residents. Geriatr Nurs.

[5] Lin LC, Wu SC, Chen HS, Wang TG, Chen MY (2002). Prevalence of impaired swallowing in institutionalized older people in Taiwan. J Am Geriatr Soc.

[6] Sura L, Madhavan A, Carnaby G, Crary MA (2012). Dysphagia in the elderly: management and nutritional considerations. Clin Interv Aging.

[7] Mann G, Hankey GJ, Cameron D (2000). Swallowing disorders following acute stroke: prevalence and diagnostic accuracy. Cerebrovasc Dis.

[8] Bergman H, Ferrucci L, Guralnik J, Hogan DB, Hummel S, Karunananthan S, Wolfson C (2007). Frailty: an emerging research and clinical paradigm-issues and controversies. J Gerontol A Biol Sci Med Sci.

[9] Logemann JA, Gensler G, Robbins J, Lindblad AS, Brandt D, Hind JA, Kosek S, Dikeman K, Kazandjian M, Gramigna GD, Lundy D, McGarvey-Toler S, Miller Gardner PJ (2008). A randomized study of three interventions for aspiration of thin liquids in patients with dementia or Parkinson’s disease. J Speech Lang Hear Res.

[10] Hickson M, Frost G (2004). An investigation into the relationship between quality of life, nutritional status and physical function. Clin Nutr.

[11] Serra-Prat M, Palomera M, Gomez C, Sar-Shalom D, Saiz A, Montoya JG, Navajas M, Palomera E, Clave P (2012). Oropharyngeal dysphagia as a risk factor for malnutrition and lower respiratory tract infection in independently living older persons: a population-based prospective study. Age Aging.

[12] Stookey JD, Pieper CF, Cohen HJ (2005). Is the prevalence of dehydration among community-dwelling older adults really low? Informing current debate over the fluid recommendation for adults aged 70+ years. Public Health Nutr.

[13] Morley JE (2015). Dysphagia and aspiration. J Am Med Dir Assoc.

[14] Wirth R, Pourhassan M, Streicher M, Hiesmayr M, Schindler K, Sieber CC, Volkert D (2018). The impact of dysphagia on mortality of nursing home residents: results from the NutritionDay project. J Am Med Dir Assoc.

[15] Verdonschot RJ, Baijens LW, Serroyen JL, Leue C, Kremer B (2013). Symptoms of anxiety and depression assessed with the hospital anxiety and depression scale in patients with oropharyngeal dysphagia. J Psychosom Res.

[16] Ebihara S, Sekiya H, Miyagi M, Ebihara T, Okazaki T (2016). Dysphagia, dystussia, and aspiration pneumonia in elderly people. J Thorac Dis.

[17] Smith CH, Logemann JA, Colangelo LA, Rademaker AW, Pauloski BR (1999). Incidence and patient characteristics associated with silent aspiration in the acute care setting. Dysphagia.

[18] Garon BR, Sierzant T, Ormiston C (2009). Silent aspiration: results of 2,000 video fluoroscopic evaluations. J Neurosci Nurs.

[19] Nobrega AC, Rodrigues B, Melo A (2008). Is silent aspiration a risk factor for respiratory infection in Parkinson’s disease patients. Parkinsonism Relat Disord.

[20] Cabre M, Serra-Prat M, Palomera E, Almirall J, Pallares R, Clave P (2010). Prevalence and prognostic implications of dysphagia in elderly patients with pneumonia. Age Ageing.

[21] Almirall J, Cabre M, Clave P (2012). Complications of oropharyngeal dysphagia: aspiration pneumonia. Nestle Nutr Inst Workshop Ser.

[22] Clave P, Rofes L, Carrion S, Ortega O, Cabre M, Serra-Prat M (2012). Pathophysiology, relevance and natural history of oropharyngeal dysphagia among older people. Nestle Nutr Inst Workshop Ser.

[23] Peters MDJ, Godfrey C, McInerney P, Baldini Soares C, Khalil H, Parker D, Aromataris E, Munn Z (2017). Chapter 11: Scoping Reviews. Joanna Briggs Institute Reviewer's Manual. The Joanna Briggs Institute.

[24] Tricco AC, Lillie E, Zarin W, O'Brien KK, Colquhoun H, Levac D, Moher D, Peters MDJ, Horsley T, Weeks L, Hempel S, Akl EA, Chang C, McGowan J, Stewart L, Hartling L, Aldcroft A, Wilson MG, Garritty C, Lewin S, Godfrey CM, Macdonald MT, Langlois EV, Soares-Weiser K, Moriarty J, Clifford T, Tunçalp Ö, Straus SE (2018). PRISMA extension for scoping reviews (PRISMA-ScR): checklist and explanation. Ann Intern Med.

[25] Cormary X, Tannou Y, Bras M, Culis M, Cugy E, Blasco-Baque V (2018). Pleasure of eating despite dysphagia in nursing homes : a multi-disciplinary plan. Dysphagia.

[26] Freiry AM, De Matos DM, Haack BG, Olchik MR, Ghisi M (2017). Efficacy of an intervention program in elderly with dysphagia and dementia resident long-term institution: A series of cases. Int Arch Otorhinolaryngol.

[27] Gilmore-Bykovskyi AL, Rogus-Pulia N (2018). Temporal associations between caregiving approach, behavioral symptoms and observable indicators of aspiration in nursing home residents with dementia. J Nutr Health Aging.

[28] Gokula M, Rubeen S, Thotakura S (2011). Does amantadine help elderly residents with symptomless dysphagia?. Ann Long-Term Care.

[29] Hagglund P, Hagg M, Wester P, Levring JE (2019). Effects of oral neuromuscular training on swallowing dysfunction among older people in intermediate care-a cluster randomised, controlled trial. Age Ageing.

[30] Hajjar S, Wollman D. Resident presentation dysphagia in elderly. J Am Geriatr Soc. 2019;67, S278(1):–S279.

[31] Kuramoto N, Jayatilake D, Hidaka K, Suzuki K (2018). Automatic measurements of neck flexion using smartphone-based swallowing monitor. Dysphagia.

[32] Levenson SA, Walker VL (2019). It is time to revamp approaches to managing dysphagia in nursing homes. J Am Med Dir Assoc.

[33] Luk JKH, Chan DKY (2014). Preventing aspiration pneumonia in older people: do we have the 'know-how'?. Hong Kong Medical Journal.

[34] Mesioye A, Smith J, Zilberstein M, Hart H, Bush-Thomas P, Ward M (2018). Dysphagia rounds: Interdisciplinary collaboration to improve swallowing safety in a VA community living center. J Am Geriatr Soc.

[35] Hines S, McCrow J, Abbey J, Gledhill S (2010). Thickened fluids for people with dementia in residential aged care facilities. Int J Evid Based Healthcar.

[36] Painter V, Le Couteur DG, Waite LM (2017). Texture-modified food and fluids in dementia and residential aged care facilities. Clin Interv Aging.

[37] Park Y, Oh S, Chang H, Bang HL (2015). Effects of the evidence-based nursing care algorithm of dysphagia for nursing home residents. J Gerontol Nurs.

[38] Richards E (2012). Communication and swallowing problems after stroke. Nurs Residential Care.

[39] Yamada T, Matsuo K, Izawa M, Yamada S, Masuda Y, Ogasawara T (2017). Effects of age and viscosity on food transport and breathing-swallowing coordination during eating of two-phase food in nursing home residents. Geriatr Gerontol Int.

[40] Lu M, Hong L, He LM, Gao F, Xu J, Jiang LN (2018). Effect of community nursing intervention in elderly people with dysphagia in nursing homes. Shanghai Med Pharm J.

[41] Chiang CK, Hwu YJ (2018). Feeding experiences of nursing aides for residents with dysphagia. Geriatr Nurs.

[42] Takamoto K, Saitoh T, Taguchi T, Nishimaru H, Urakawa S, Sakai S, Ono T, Nishijo H (2018). Lip closure training improves eating behaviors and prefrontal cortical hemodynamic activity and decreases daytime sleep in elderly persons. J Bodyw Mov Ther.

[43] Chen S, Cui Y, Ding Y, Sun C, Xing Y, Zhou R, Liu G (2020). Prevalence and risk factors of dysphagia among nursing home residents in eastern China: a cross-sectional study. BMC Geriatr.

[44] Ministry of Civil Affairs of the People’s Republic of China (2018). Social services development statistical communique in 2017.

[45] Beverly C, Burger SG, Maas ML, Specht JK (2010). Aging issues: nursing imperatives for healthcare reform. Nurs Adm Q.

[46] Beck AM, Kjaersgaard A, Hansen T, Poulsen I (2018). Systematic review and evidence based recommendations on texture modified foods and thickened liquids for adults (above 17 years) with oropharyngeal dysphagia-an updated clinical guideline. Clin Nutr.

[47] O'Keeffe ST (2018). Use of modified diets to prevent aspiration in oropharyngeal dysphagia: is current practice justified?. BMC Geriatr.

[48] Carlisle BJ, Craft G, Harmon JP, Ilkevitch A, Nicoghosian J, Sheyner I, Stewart JT (2016). PEG and Thickeners: a critical interaction between polyethylene glycol laxative and starch-based Thickeners. J Am Med Dir Assoc.

[49] Ebihara S, Ebihara T (2011). Cough in the elderly: a novel strategy for preventing aspiration pneumonia. Pulm Pharmacol Ther.

[50] Logemann JA, Gensler G, Robbins J, Lindblad AS, Brandt D, Hind JA, Kosek S, Dikeman K, Kazandjian M, Gramigna GD, Lundy D, McGarvey-Toler S, Miller Gardner PJ (2008). A randomized study of three interventions for aspiration of thin liquids in patients with dementia or Parkinson's disease. J Speech Lang Hear Res.

[51] Terré R, Mearin F (2012). Effectiveness of chin-down posture to prevent tracheal aspiration in dysphagia secondary to acquired brain injury: a videofluoroscopy study. Neurogastroenterol Motil.

[52] Solazzo A, Monaco L, Del Vecchio L, Tamburrini S, Iacobellis F, Berritto D (2012). Investigation of compensatory postures with videofluoromanometry in dysphagia patients. World J Gastroenterol.

[53] Rofes L, Arreola V, Almirall J, Cabré M, Campins L, García-Peris P (2011). Diagnosis and management of oropharyngeal dysphagia and its nutritional and respiratory complications in the elderly. Gastroenterol Res Pract.

[54] Sze WP, Yoon WL, Escoffier N, Rickard Liow SJ (2016). Evaluating the training effects of two swallowing rehabilitation therapies using surface electromyography-Chin Tuck against resistance (CTAR) exercise and the shaker exercise. Dysphagia..

[55] Cabre M, Serra-Prat M, Palomera E, Almirall J, Pallares R, Clavé P (2010). Prevalence and prognostic implications of dysphagia in elderly patients with pneumonia. Age Ageing.

[56] Rofes L, Arreola V, López I, Martin A, Sebastián M, Ciurana A, Clavé P (2013). Effect of surface sensory and motor electrical stimulation on chronic poststroke oropharyngeal dysfunction. Neurogastroenterol Motil.

[57] Olchik MR, Rech RS, Jacinto-Scudeiro LA, Mello AM, Santos VB (2020). The effects of orofacial thermal-tactile stimulation on elderly long-term-care facility residents with severe dementia: a case series. Audiol Commun Res.

[58] Waldron C, Nunn J, Mac Giolla Phadraig C, Comiskey C, Guerin S, van Harten MT (2019). Oral hygiene interventions for people with intellectual disabilities. Cochrane Database Syst Rev.

